# Schisandrin A Inhibits the IL-1β-Induced Inflammation and Cartilage Degradation via Suppression of MAPK and NF-κB Signal Pathways in Rat Chondrocytes

**DOI:** 10.3389/fphar.2019.00041

**Published:** 2019-01-29

**Authors:** Chang Tu, Xiaojian Huang, Yifan Xiao, Mingyu Song, Yongzhuang Ma, Jiyuan Yan, Hongbo You, Hua Wu

**Affiliations:** ^1^Department of Orthopedics, Tongji Hospital, Tongji Medical College, Huazhong University of Science and Technology, Wuhan, China; ^2^Department of Immunology, School of Basic Medicine, Tongji Medical College, Huazhong University of Science and Technology, Wuhan, China; ^3^Department of Obstetrics and Gynecology, Tongji Hospital, Tongji Medical College, Huazhong University of Science and Technology, Wuhan, China

**Keywords:** Schisandrin A, osteoarthritis, iNOS, Cox-2, MMPs, ADAMTS5, MAPK, NF-κB

## Abstract

Osteoarthritis (OA) is a common joint disease in the elderly population. Its development has been reported to be associated with cartilage degradation and inflammatory responses. Schisandrin A, a bioactive lignin in *Schisandra sphenanthera*, has shown its anti-inflammatory potential in various inflammation diseases. However, the effects of Schisandrin A on OA remain to explore. In this study, rat chondrocytes were treated with IL-1β (10 ng/ml) with or without different concentrations of Schisandrin A for 24 h. Cell viability was evaluated by CCK-8 assay. Production of nitric oxide (NO) and prostaglandin E2 (PGE2) was measured by the Griess reaction and ELISA. The MAPK/NF-κB-related signaling molecules expression and the protein production of inducible nitric oxide synthase (iNOS), cyclooxygenase (Cox)-2, MMPs (MMP1, MMP3, MMP13), ADAMTS5, Collagen II, aggrecan, and Sox9 were detected by Western blot. Protein expression of Collagen II, aggrecan, and p65 nuclear translocation was evaluated by immunofluorescence. *In vivo*, intra-articular injection of 50 μM Schisandrin A or equal volume of vehicle was performed on rat OA models. Severity of cartilage damage was evaluated by HE and Safranin-O-Fast green staining. Our results revealed that Schisandrin A could suppress the IL-1β-induced production of NO and PGE2 in rat chondrocytes. Consistent with these findings, the upregulation of iNOS and Cox2 could also been decreased by Schisandrin A. Additionally, Schisandrin A could inhibit IL-1β-induced cartilage matrix catabolic enzymes including MMPs and ADAMTS5. Moreover, the IL-1β-induced downregulation of Collagen II, aggrecan, and Sox9 could be ameliorated by Schisandrin A. Mechanistically, Schisandrin A functioned by suppressing MAPK and NF-κB signal pathways. *In vivo*, Schisandrin A prevented cartilage damage in rat OA model. In conclusion, this study elucidates that Schisandrin A inhibits the IL-1β-induced inflammation and cartilage degradation via suppression of MAPK and NF-κB signal pathways, indicating its potential role in OA therapy.

## Introduction

Osteoarthritis (OA) is a chronic joint disease characterized by cartilage degeneration and physical disability (Jeon et al., 2017). Hallmarks of OA include arthrodynia, osteophyte formation, and subchondral bone sclerosis (Glyn-Jones et al., 2015). Pervasive cartilage damage due to inflammation and imbalance between anabolic and catabolic factors in joint is essential to the progression of OA (Appleton, 2018). As an age-related disease, OA affects 240 million people globally with about 10% of men and 18% of women over 60 years old (Nelson, 2018). It is confirmed that OA has doubled in prevalence since the mid-20th century (Wallace et al., 2017). However, due to lack of effective treatment for OA, most patients finally need joint replacement, leaving behind huge social and economic burdens (Bellamy et al., 2006).

Reportedly, local inflammation responses play important role in the development of OA (Bonnet and Walsh, 2005). High level of proinflammatory cytokine interleukin-1β (IL-1β) was detected in the synovial fluid during OA (Dodge and Poole, 1989). In chondrocytes, IL-1β can up-regulate the expression of metalloproteinases (MMPs), aggrecanase-2 (ADAMTS5), and down-regulate the synthesis of extracellular matrix components Collagen II and aggrecan (Jacques et al., 2006). Moreover, IL-1β has been shown to induce the overproduction of inducible nitric oxide synthase (iNOS) and cyclooxygenase-2 (COX-2), which is directly relevant to the secretion of nitric oxide (NO) and prostaglandin E2 (PGE2) (Sheu et al., 2015). Strategies targeting IL-1β may provide breakthrough in OA treatment.

Schisandrin A, one of the major bioactive components of *Schisandra sphenanthera*, has been indicated to play a role in the anti-inflammatory and anti-oxidative activities (Liang et al., 2014; Song et al., 2016). Meanwhile, a recent study revealed that Schisandrin A could notably suppress the activation of MAPK and NF-κB pathways induced by lipopolysaccharide in RAW 264.7 macrophages, suggesting the underlying mechanisms of its anti-inflammatory properties (Kwon et al., 2018). Moreover, an *in vivo* research has shown that the protective effects of Schisandrin A on acute liver injury (Lu et al., 2014). However, the exact role of Schisandrin A in OA remains unclear. In this study, the anti-inflammatory effects and mechanism of Schisandrin A on IL-1β-induced rat chondrocytes were investigated. We expect to explore a promising pharmacological target in OA therapy.

## Materials and Methods

### Ethics Statement

This study was carried out in accordance with the Guidelines of Animal Care and Use Committee for Teaching and Research, Tongji Medical College, Huazhong University of Science and Technology. The protocol was approved by the Institutional Animal Care and Use Committee, Tongji Medical College, Huazhong University of Science and Technology. All efforts were made to minimize animal suffering.

### Regents

Schisandrin A, dimethylsulfoxide (DMSO) and collagenase II were obtained from Sigma-Aldrich (St. Louis, MO, United States). Schisandrin A was dissolved in DMSO and stored at −80°C. Control group was added with DMSO (Vehicle) in the cell experiments. Recombinant rat IL-1β (501-RL-010) and PGE2 ELISA kit were procured from R&D Systems (Minneapolis, MN, United States). Dulbecco’s modified Eagle’s medium F12 (DMEM/F12) was purchased from HyClone (Grand Island, NY, United States). Antibodies against MMP1, p65, Collagen II, and IκBα were provided from Proteintech Group (Wuhan, China). Antibodies specific for MMP13 and iNOS were purchased from Abcam (Cambridge, United Kingdom). Antibodies against COX2, MMP3, p-p65, p-IκBα, ERK1/2, p-ERK, p38, p-p38, JNK, p-JNK were supplied by Cell Signaling Technology (Beverly, MA, United States). Antibodies against Aggrecan was acquired from Santa Cruz Biotechnology (Santa Cruz, CA, United States). Antibodies specific for GAPDH, ADAMTS5 and secondary antibodies were procured from Boster (Wuhan, China).

### Cell Culture

Five days old Sprague-Dawley (SD) rats were procured from the Laboratory Animal Center of Tongji hospital of Hubei province in China. All experimental procedures were complying with the Guidelines of Animal Care and Use Committee for Teaching and Research of Huazhong University of Science and Technology. Rat chondrocytes were isolated as described preciously (Oh et al., 2016). Briefly, cartilage acquired from the bilateral knee joint was minced into small pieces. Then pieces were digested primarily with 0.25% trypsin-EDTA solution at 37°C for 30 min and subsequently with 0.25% collagenase II in DMEM/F12 at 37°C for 8 h. Cell suspension was centrifugated (1200 rpm for 5 min) to collect the chondrocytes. Isolated chondrocytes were cultured in DMEM/F12 containing 10% fetal bovine serum (FBS, Gibco, NY, United States), 100 U/ml penicillin and 100 U/ml streptomycin (Sigma-Aldrich, St. Louis, MO, United States) at 37°C with 5% CO_2_. The second or third passages were used in the following experiments.

### Cell Viability

Chondrocytes were seeded in 96-well plates at a density of 1 × 10^4^/well. The concentration range of Schisandrin A used in this assay was based on previous study (Song et al., 2016). Cells were cultured with various concentrations of Schisandrin A in the absence or presence of IL-1β (10 ng/ml) for 24 h. Subsequently, cell viability was measured with a cell counting kit-8 (CCK-8, Boster, Wuhan, China) following the standard protocol. Briefly, 100 μl culture medium containing 10 μl CCK-8 solution was added into each well. After 1 h incubation at 37°C with 5% CO_2_, the absorbance at 450 nm was detected using a microplate reader (Bio-Rad, Richmond, CA, United States).

### NO and PGE2 Measurement

To examine the levels of NO and PGE2, chondrocytes were exposed to IL-1β (10 ng/ml) with or without different concentrations (25 and 50 μM) of Schisandrin A. Cell culture supernatants were harvested and stored in −80°C. Griess reaction was performed to measure the NO concentration and PGE2 level was detected with an ELISA kit following the manufacturer’s protocol. All assays were performed in triplicate.

### Western Blot Analysis

Chondrocytes were washed with PBS three times and lysed with RIPA supplemented with 1 mM protease inhibitor cocktail and 1 mM phosphatase inhibitor cocktail (Boster, Wuhan, China). Twenty five micrograms protein samples were separated on SDS-polyacrylamide gels and transfered onto a PVDF membrane. The membrane was firstly blocked with 5% bovine serum albumin (BSA) for 1 h and then incubated overnight with primary antibodies at 4°C. Subsequently, blots were washed with Tris-buffered saline with 0.1% Tween-20 (TBST) three times and incubated with horse-radish peroxidase (HRP)-conjugated secondary antibodies for 1 h at room temperature. Finally, the immunoreactive bands were detected with the Western ECL System (Boster, Wuhan, China). GAPDH was used as the loading control and representative bands were shown. Western blots were repeated at least three times.

### Immunofluorescence

Chondrocytes were seeded at a density of 1 × 10^4^ cells/well in 24 well plates. Cells were treated with 10 ng/ml IL-1β in the absence or presence of 50 μM Schisandrin A. After fixation with 4% paraformaldehyde for 15 min at room temperature, the cells were permeabilized with PBS containing 0.2% Triton X-100 for 5 min and blocked with 5% BSA for 30 min. Cells were then incubated with antibodies specific for Collagen II, aggrecan and P65 overnight at 4°C. Afterward, the cells were washed three times with PBS and incubated with Cy3-conjugated secondary antibodies for 1 h at 37°C in the dark. Finally, cell nucleuses were stained with DAPI for 10 min. A fluorescence microscope (Evos flauto, Life Technologies, United States) was used to acquire the images.

### Animal Experiment Design

Twenty-four 6-week-old male SD rats (about 220–260 g) were provided by the Laboratory Animal Center of Tongji Hospital and were approved by the Committee. Rat OA models were built by open surgery involving anterior cruciate ligament transection (ACL-T) and partial medial meniscectomy as described before (Appleton et al., 2007). Briefly, Surgery was conducted only on the right knee. After anesthetizing with pentobarbital (3.5 mg/100 g weight) injection intraperitoneally, an incision between the medial aspect of the joint capsule and medial collateral ligament was made. Then, ACL and part of the medial meniscus were removed without any cartilage or ligament damage. Afterward, the joint capsule and the skin were sutured, respectively, and the rat OA model was finished. For the control group, rats were only performed the same incision without meniscus or ACL resection. For the Schisandrin A group, rat was performed intra-articular injection of 50 μM Schisandrin A weekly after surgery. For the vehicle group, rat was performed intra-articular injection of equal volume of vehicle as the Schisandrin A group. One month post surgery, all animals were sacrificed and samples were photographed and fixed with 4% paraformaldehyde for further analysis.

### Histological Evaluation

Fixed samples were decalcified and embedded in paraffin. Then the knee joints were cut coronally, stained with hematoxylin and eosin (H&E) and Safranin-O-Fast green, and photographed with an inverted microscope. The Osteoarthritis Research Society International (OARSI) scoring system was used to quantified the histological evaluation (Pritzker et al., 2006).

### Statistical Analysis

Values were shown as the mean ± standard deviation (SD). Data comparisons were analyzed by one-way analysis of variance (ANOVA) followed by Tukey *post hoc* test. Significance was confirmed at *P* < 0.05. All experiments were repeated three times.

## Results

### Effects of Schisandrin A on Cell Viability

**FIGURE 1:**
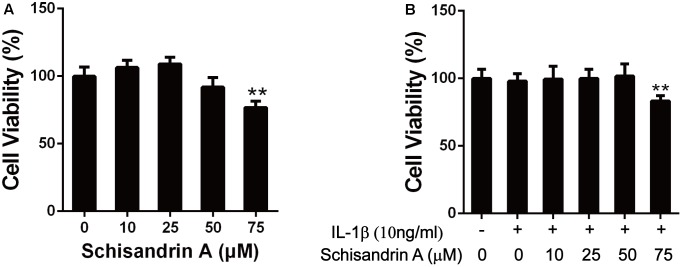
Effects of Schisandrin A on cell viability. **(A)** Rat chondrocytes were exposed to Schisandrin A (25, 50 μM) alone or **(B)** with IL-1β (10 ng/ml) for 24 h and cell viability was determined by CCK-8 assay. ^∗∗^*P* < 0.01 vs. control group.

CCK-8 assay was employed to verify the cytotoxic effects of Schisandrin A on chondrocytes. As shown in Figure 1, Schisandrin A at the concentrations 10, 25, and 50 μM exhibited no cytotoxicity on chondrocytes at 24 h with or without IL-1β (10 ng/ml). However, cell viability was decreased with the treatment of 75 μM Schisandrin A. Therefore, Schisandrin A with concentrations of 25 and 50 μM was used for subsequent experiments.

### Effects of Schisandrin A on IL-1β-Induced Production of NO, PGE2, iNOS, and COX2 in Rat Chondrocytes

**FIGURE 2:**
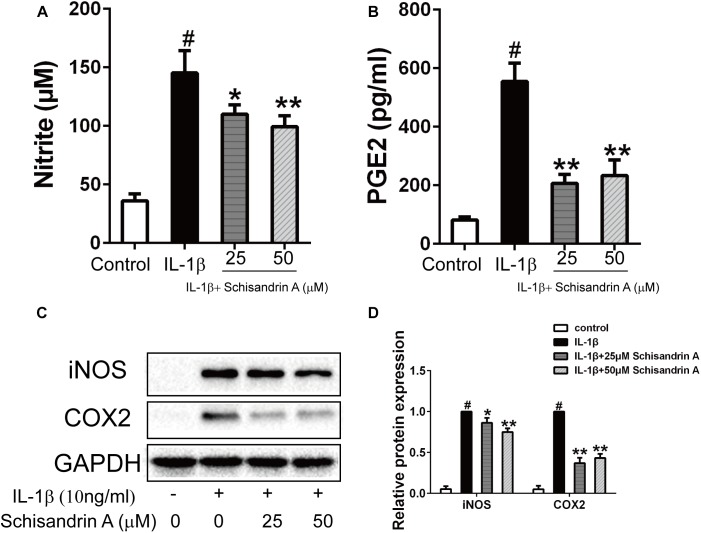
Effects of Schisandrin A on IL-1β-induced production of NO, PGE2, iNOS, and COX2 in rat chondrocytes. Cells were treated with Schisandrin A (25, 50 μM) in the presence or absence of IL-1β (10 ng/ml) for 24 h. Cell culture supernatants were collected. **(A)** Griess reaction was used to measure the NO concentration (*n* = 3). **(B)** PGE2 level was accessed by ELISA (*n* = 3). **(C)** Expression of iNOS and COX2 were detected by Western blot. **(D)** Relative protein expression was qualified by ImageJ software, GAPDH was served as the loading control (*n* = 3). ^#^*P* < 0.05 vs. control group; ^∗^*P* < 0.05 vs. IL-1β group; ^∗∗^*P* < 0.01 vs. IL-1β group.

To verify whether Schisandrin A could inhibit the upregulation of NO and PGE2 induced by IL-1β, the Griess reagent was performed to detect the NO concentration and an ELISA kit was used to evaluate the PGE2 level. Schisandrin A at 25 and 50 μM could markedly suppress the production of NO and PGE2 (Figure 2A,B). Western blot was used to further confirm the effects of Schisandrin A on the expression of iNOS and COX2. Chondrocytes were exposed to IL-1β with or without different concentrations of Schisandrin A for 24 h. Consequently, similar concentrations of Schisandrin A could reduce the increased protein production of iNOS and COX2 induced by IL-1β (Figure 2C,D).

### Effects of Schisandrin A on IL-1β-Induced Protein Expression of MMPs and ADAMTS5 in Rat Chondrocytes

**FIGURE 3:**
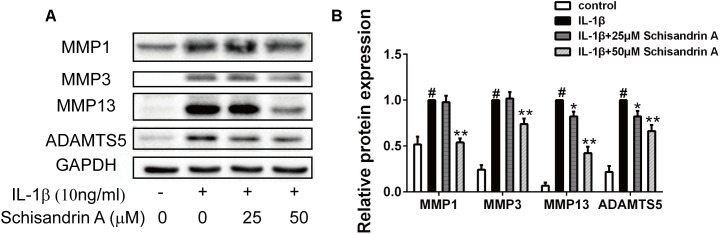
Effects of Schisandrin A on IL-1β-induced MMPs and ADAMTS5 protein expression. Chondrocytes were exposed to Schisandrin A (25, 50 μM) with or without IL-1β (10 ng/ml) for 24 h. **(A)** Western blot was employed to determine the expression of MMP1, MMP3, MMP13, and ADAMTS5. **(B)** Relative protein expression was qualified by Image-J software, GAPDH was used as the internal control (*n* = 3). ^#^*P* < 0.05 vs. control group; ^∗^*P* < 0.05 vs. IL-1β group; ^∗∗^*P* < 0.01 vs. IL-1β group.

MMPs (MMP1, MMP3, MMP13) and ADAMTS5 are the major cartilage degradation enzymes during the progress of OA. In this study, chondrocytes were treated with Schisandrin A in the presence or absence of IL-1β for 24 h. As indicated in Figure 3, IL-1β could markedly upregulate the expression of MMPs and ADAMTS5 in chondrocytes. However, 50 μM Schisandrin A could effectively reverse the upregulation of MMP1 and MMP3. Furthermore, Schisandrin ameliorated IL-1β-induced protein upregulation of MMP13 and ADAMTS5 in a concentration–dependent manner.

### Effects of Schisandrin A on IL-1β-Induced Degradation of Rat Cartilage

**FIGURE 4:**
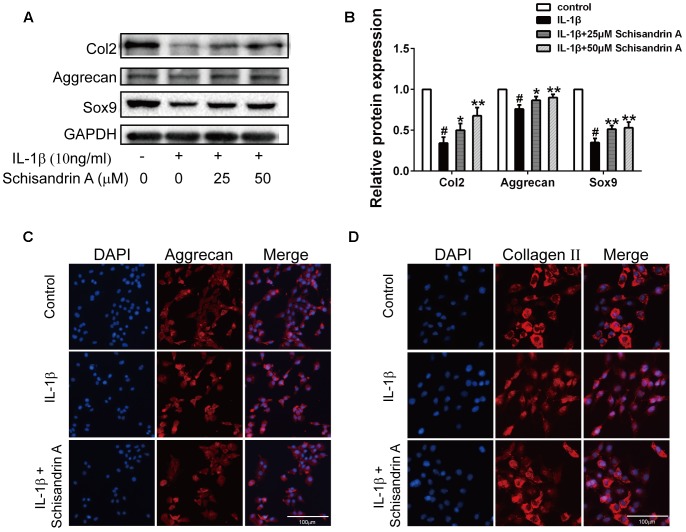
Effects of Schisandrin A on IL-1β-induced cartilage degradation. **(A)** Cells were treated with Schisandrin A (25, 50 μM) in the presence or absence of IL-1β (10 ng/ml) for 24 h. Protein levels of Collagen II, Aggrecan and Sox9 were determined by Western Blot. **(B)** Relative protein expression was qualified by Image-J software, GAPDH was used as the loading control (*n* = 3). **(C)** Aggrecan and **(D)** Collagen II were observed by Immunofluorescence after cells were treated with IL-1β (10 ng/ml) with or without Schisandrin A (50 μM) for 24 h. ^#^*P* < 0.05 vs. control group; ^∗^*P* < 0.05 vs. IL-1β group; ^∗∗^*P* < 0.01 vs. IL-1β group.

Collagen II, Aggrecan and Sox9 are commonly used to evaluate cartilage degradation. Chondrocytes were exposed to IL-1β with or without Schisandrin A for 24 h. Western blot and Immunofluorescence were employed to detect the expression of Collagen II, aggrecan, and Sox9. As shown in Figure 4A,B, Schisandrin A could dose-dependently reverse the IL-1β-induced downregulation of Collagen II, Aggrecan, and Sox9 in protein levels. Furthermore, the fluorescent result exhibited the same tendency (Figure 4C,D).

### Effects of Schisandrin A on IL-1β-Induced MAPK Signal Activation in Rat Chondrocytes

**FIGURE 5:**
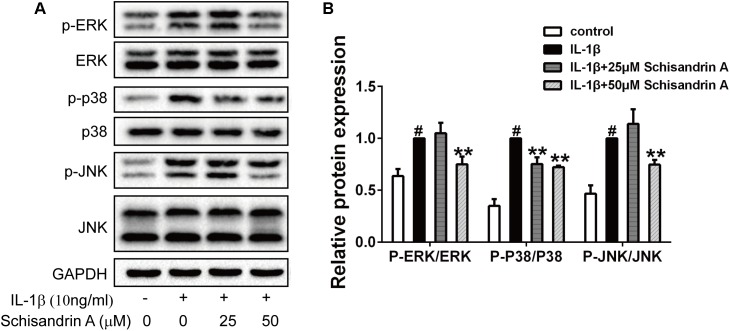
Effects of Schisandrin A on MAPK signaling pathway. Rat chondrocytes were exposed to Schisandrin A (25, 50 μM) with or without IL-1β (10 ng/ml) for 30 min. **(A)** Phosphorylations of ERK, p38, JNK were determined by Western blot. **(B)** Relative protein expression was qualified by ImageJ software, ERK, p38, and JNK were used as the internal control, respectively (*n* = 3). ^#^*P* < 0.05 vs. control group; ^∗∗^*P* < 0.01 vs. IL-1β group.

MAPK signal pathway is highly associated with the progress of OA. Chondrocytes were exposed to IL-1β with or without Schisandrin A for 30 min. Western blot analysis was used to detect the phosphorylations of ERK, p38 and JNK. As shown in Figure 5, IL-1β treatment significantly increased the p-ERK, p-p38, p-JNK levels. By contrast, 50 μM Schisandrin A could effectively suppress the activation of ERK and JNK pathways. Moreover, Schisandrin A inhibited the activation of p38 pathway in a concentration–dependent manner.

### Effects of Schisandrin A on IL-1β-Induced NF-κB Signal Activation in Rat Chondrocytes

**FIGURE 6:**
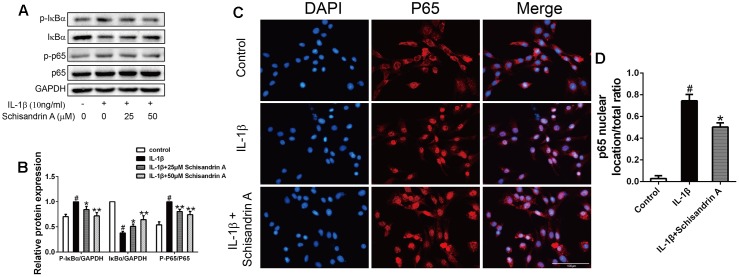
Effects of Schisandrin A on NF-κB signaling pathway. Cells were exposed to Schisandrin A (25, 50 μM) with or without IL-1β (10 ng/ml) for 30 min. **(A)** Protein levels of p-IκBα, IκBα, p-p65, p65 were detected by Western blot. **(B)** Relative protein expression was qualified by ImageJ software, GAPDH and p65 were used as the loading control, respectively (*n* = 3). **(C)** p65 translocation was observed by Immunofluorescence. **(D)** Quantitative analysis of p65 nuclear location/total ratio of three groups. ^#^*P* < 0.05 vs. control group; ^∗^*P* < 0.05 vs. IL-1β group; ^∗∗^*P* < 0.01 vs. IL-1β group.

Activation of NF-κB plays crucial roles in the inflammation responses and cartilage degradation of OA. In our study, chondrocytes were treated with Schisandrin A in the presence or absence of IL-1β for 30 min. Western blot was employed to detect the activation of NF-κB and degradation of IκBα. As shown in Figure 6A,B, treatment of IL-1β markedly induced degradation and phosphorylation of IκBα, and the upregulation of p-p65. However, this trend can be partly restored by Schisandrin A in a concentration–dependent manner. Further, 50 μM Schisandrin A could significantly suppress the IL-1β-induced nuclear translocation of p65 in chodrocytes (Figure 6C,D).

### Effects of Schisandrin A on Rat OA Model

To manifest the effects of Schisandrin A on rat OA *in vivo*, we constructed rat OA models by removing ACL and partial medial meniscus. After a month of surgery, knee joint samples of three groups were harvested. All animals recovered from the surgery and no complications or infections were observed.

**FIGURE 7:**
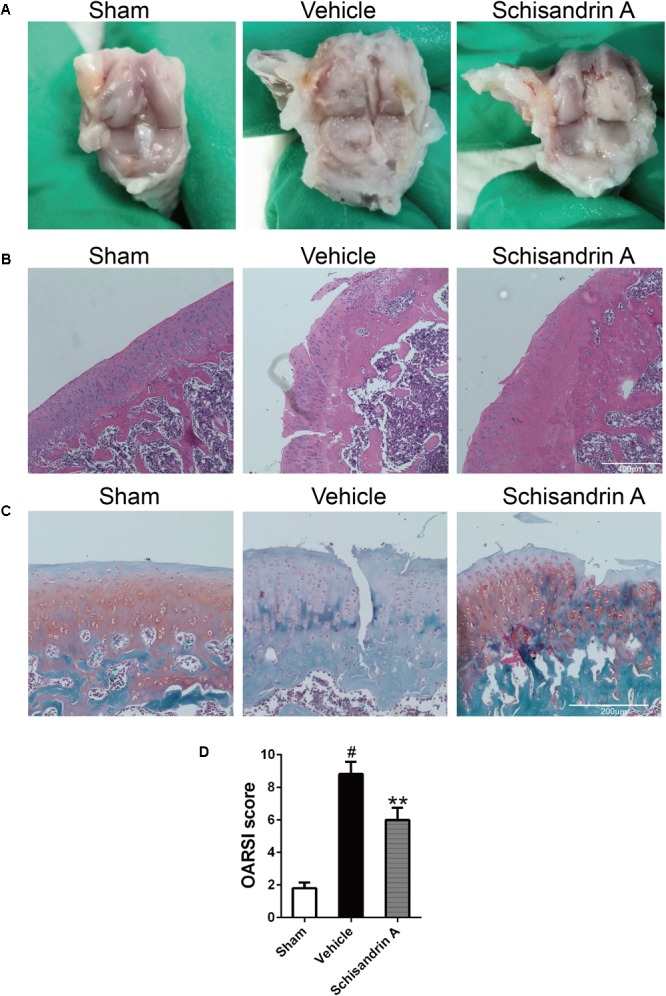
Effects of Schisandrin A on rat Osteoarthritis. **(A)** Marcroscopic images of rat knee joint samples of three groups. **(B)** Microscopic photos of HE stained rat knee joint sections of three groups. **(C)** Microscopic photos of Safranin-O-Fast green stained rat knee joint sections of three groups. **(D)** The OARSI scores of each group. ^#^*P* < 0.05 vs. sham group; ^∗∗^*P* < 0.01 vs. vehicle group.

For gross study, knee joint from the sham group exhibited the normal structure with smooth and glassy cartilage. By contrast, severe surface abrasions and tissue fibrosis were observed in the vehicle group. Intra-articular injection of 50 μM Schisandrin A could alleviate joint damage according to the comparison between vehicle and Schisandrin A groups (Figure 7A).

For histological evaluation, samples of each group were stained by HE and Safranin-O-Fast green. Normal structure of cartilage was observed in sham group. Cartilage damage characterized by fissuring in matrix, disorganized sequence of chondrocytes and loss of Safranin-O staining was seen in vehicle group (Figure 7B,C). By comparing the OARSI scores of the three groups, 50 μM Schisandrin A treatment could effectively reverse the cartilage damage in OA (Figure 7D).

## Discussion

**FIGURE 8:**
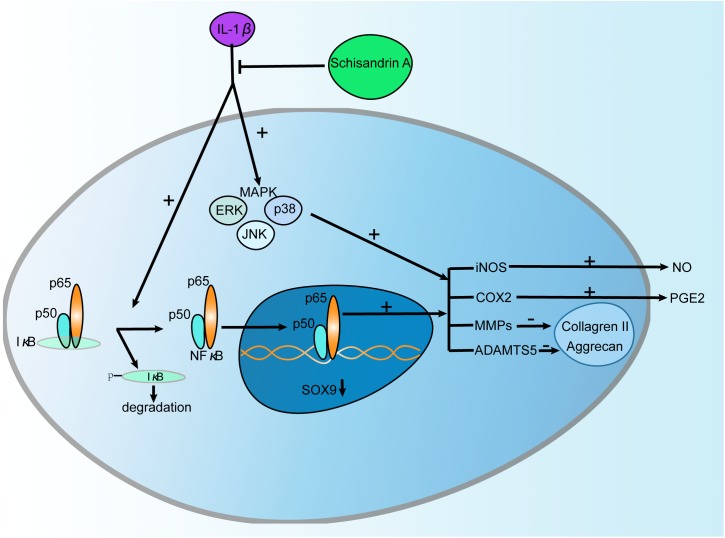
Schematic diagram of the effect of Schisandrin A on IL-1β-Induced Inflammation and Cartilage Degradation. IL-1β induces proinflammatory factors including iNOS, COX2, MMPs, and ADAMTS5 which subsequently trigger the release of NO, PGE2 and degradation of Collageren II, Aggrecan. Further, IL-1β functions by activating MAPK and NF-κB signaling pathways characterized by upregulation of p-ERK, p-p38, p-JNK, p-IκBα, p-p65 and degradation of IκBα, and translocation of p65. However, Schisandrin A can reverse this process.

Osteoarthritis is an age-associated musculoskeletal disease featured by cartilage degeneration, abnormal bone adaptations, and subchondral bone sclerosis (Panahifar et al., 2014). Current strategies for OA are faced with unmet success. Therapies for OA focus mainly on symptom relief, while the pathological process is poorly unraveled (Su et al., 2014). This forces most patients with advanced OA to undergo joint replacement. Therefore, it is urgent to find effective agents for OA treatment. Schisandrin A, a bioactive component of *S. sphenanthera*, has earned its reputations for anti-inflammatory potential in various diseases including neuroinflammation (Song et al., 2016), acute liver injury (Lu et al., 2014), and myocardial ischemia-reperfusion injury (Chang et al., 2013). In this study, we are the first to report the therapeutic effects of Schisandrin A on the IL-1β-induced inflammation and cartilage degradation via suppression of MAPK and NF-κB signal pathways in rat chondrocytes (Figure 8).

IL-1β as a proinflammatory cytokine plays a crucial role in OA (Fernandes et al., 2002). OA development is associated with local inflammation responses and disorders of anabolic and metabolic process. Previous study confirmed that IL-1β induced increased OA-related protein expressions in a time and concentration dependent manner. Accordingly, IL-1β at a concentration of 10 ng/ml exhibited the strongest effect (Wang et al., 2018). So we chose 10 ng/ml IL-1β as a stimulus *in vitro*. Our data revealed that IL-1β could markedly upregulate the expression of inflammatory factors including iNOS, COX2, NO, and PGE2. Furthermore, IL-1β significantly induced the synthesis of cartilage matrix degrading enzymes such as MMP1, MMP3, MMP13 and ADAMTS5. Meanwhile, matrix synthesizing related proteins including Collagen II, Aggrecan and Sox9 were notably downregulated by IL-1β. However, all these changes above could be restored by Schisandrin A. Taken together, Schisandrin A showed anti-inflammatory and anti-degenerative effects on OA *in vitro*. To manifest the therapeutic effects of Schisandrin A on OA, *in vitro* study is far from enough. We constructed rat OA models and evaluated the protective effects of Schisandrin A on cartilage degradation *in vivo*. Results from the macroscopic view and histological analysis further confirmed the promising role of Schisandrin A by reducing cartilage destruction.

MAPK and NF-κB are two key signaling pathways in the pathogenesis of OA. MAPK activation is involved in the process of cartilage degradation induced by elevated MMPs and aggrecanases (Sondergaard et al., 2010). Transcription factor NF-κB plays a critical role in various inflammatory responses including OA (Baldwin, 2001). Due to its close connection with OA, NF-κB has been recognized as a promising target (Rigoglou and Papavassiliou, 2013). In inactive state, NF-κB presents in the cytoplasm with its inhibitor IκBα. When treated with proinflammatory cytokines such as IL-1β, NF-κB was separated from IκBα, phosphorylated, and translocated to nucleus. After nuclear translocation, it triggers the upregulaton of inflammatory factors and cartilage matrix degrading enzymes which further contribute to the progression of OA (Roman-Blas and Jimenez, 2006). In this study, IL-1β could significantly promoted the phosphorylation of ERK, p38, JNK, p65, and IκBα as well as the degradation of IκBα in rat chondrocytes. Moreover, IL-1β markedly induced the translocation of p65. However, this process could be reversed by Schisandrin A. Our observations are in accordance with other studies which reported that Schisandrin A suppressed MAPK and NF-κB signaling pathways in RAW264.7 macrophages (Kwon et al., 2018), THP-1 human monocytic cells (Guo et al., 2017a). Taken together, Schisandrin A ameliorated OA by suppressing MAPK and NF-κB signal pathways.

Schisandrin A, B, and C are the main representative lignans of *S. sphenanthera*, many studies focus on the connections and differences between them (Leong et al., 2016; Guo et al., 2017a,b). Although Schisandrin A and Schisandrin B share the close structure, their functions and mechanism of actions had been compared and studied in various diseases (Sun et al., 2014; Guo et al., 2017a). Previous study elucidated the protective effects of Schisandrin B on rat OA (Ran et al., 2018). In our research, Schisandrin A exhibited the similar anti-osteoarthritic effects, and this would further contribute to the understanding of the function of *S. sphenanthera*. Furthermore, exact target of Schisandrin A in OA remains to explore. A recent study revealed that miR-127 was involved in the protective roles of Schisandrin A on LPS-induced inflammation injury in HaCaT cells (Li et al., 2018), indicating the possible mechanism. In view of the promising therapeutic effects of Schisandrin A demonstrated by our research, further work is needed.

## Conclusion

We firstly report that Schisandrin A inhibits the IL-1β-Induced inflammation and cartilage degradation via suppression of MAPK and NF-κB signal pathways in rat chondrocytes. This study can help us to have a new understanding of the therapeutic effects of Schisandrin A on OA and provide new avenues for future OA treatment.

## Author Contributions

CT worked on conception and design. *In vivo* experiments were conducted by CT and XH. CT performed the *in vitro* experiments and wrote the paper. YX, YM, and JY contributed to the experimentation. MS, HY, and HW supervised the project, revised the manuscript, and financed the study. All authors read and approved the final manuscript.

## Conflict of Interest Statement

The authors declare that the research was conducted in the absence of any commercial or financial relationships that could be construed as a potential conflict of interest.
